# (*E*)-2-[2-(4-Fluoro­benzyl­idene)hydrazinocarbon­yl]-*N*-isopropyl­benzamide

**DOI:** 10.1107/S1600536809022181

**Published:** 2009-06-17

**Authors:** Ming Liu, Yousheng Duan, Yi Wang, Wen-Xiong Zhang, Shangzhong Liu

**Affiliations:** a Department of Applied Chemistry, China Agriculture University, 100193 Beijing, People’s Republic of China; b College of Chemistry and Molecular Engineering, Peking University, 100871 Beijing, People’s Republic of China

## Abstract

The title compound, C_18_H_18_FN_3_O_2_, adopts a *trans* conformation with respect to the C=N double bond. The dihedral angle between the two benzene rings is: 59.73 (6)°. Two independent N—H⋯O hydrogen bonds link the mol­ecules into layers parallel to (101).

## Related literature

For biologically active phthalic diamides, see: Coronado *et al.* (1994 ▶); Tohnishi *et al.* (2000 ▶). For the preparation of the title compound, see: Zaky (2002 ▶); Shigeru *et al.* (2003 ▶).
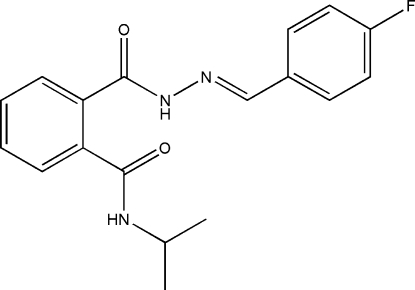


## Experimental

### 

#### Crystal data

C_18_H_18_FN_3_O_2_
*M* *_r_* = 327.35
Monoclinic, 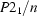

*a* = 13.316 (3) Å
*b* = 8.8904 (18) Å
*c* = 14.102 (3) Å
β = 91.10 (3)°
*V* = 1669.2 (6) Å^3^
*Z* = 4
Mo *K*α radiation
μ = 0.09 mm^−1^
*T* = 123 K
0.30 × 0.30 × 0.30 mm

#### Data collection

Rigaku R-AXIS RAPID IP diffractometer
Absorption correction: multi-scan (*ABSCOR*; Higashi,1995 ▶) *T*_min_ = 0.944, *T*_max_ = 0.972
15335 measured reflections
3833 independent reflections
2302 reflections with *I* > 2σ(*I*)
*R*_int_ = 0.046

#### Refinement

*R*[*F* ^2^ > 2σ(*F* ^2^)] = 0.039
*wR*(*F* ^2^) = 0.069
*S* = 1.02
3833 reflections
228 parameters
H atoms treated by a mixture of independent and constrained refinement
Δρ_max_ = 0.24 e Å^−3^
Δρ_min_ = −0.23 e Å^−3^

### 

Data collection: *RAPID-AUTO* (Rigaku, 2000 ▶); cell refinement: *RAPID-AUTO*; data reduction: *CrystalStructure* (Molecular Structure Corporation and Rigaku, 2000 ▶); program(s) used to solve structure: *SHELXS97* (Sheldrick, 2008 ▶); program(s) used to refine structure: *SHELXL97* (Sheldrick, 2008 ▶); molecular graphics: *SHELXTL* (Sheldrick, 2008 ▶); software used to prepare material for publication: *SHELXL97*.

## Supplementary Material

Crystal structure: contains datablocks I, global. DOI: 10.1107/S1600536809022181/ya2090sup1.cif

Structure factors: contains datablocks I. DOI: 10.1107/S1600536809022181/ya2090Isup2.hkl

Additional supplementary materials:  crystallographic information; 3D view; checkCIF report

## Figures and Tables

**Table 1:** Hydrogen-bond geometry (Å, °)

*D*—H⋯*A*	*D*—H	H⋯*A*	*D*⋯*A*	*D*—H⋯*A*
N3—H1⋯O2^i^	0.875 (15)	2.127 (15)	2.9887 (16)	168.4 (14)
N1—H2⋯O1^ii^	0.850 (15)	1.976 (15)	2.8256 (16)	177.8 (15)

Symmetry codes: (i) 
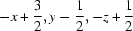
; (ii) 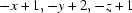.

**Fig. 1.**
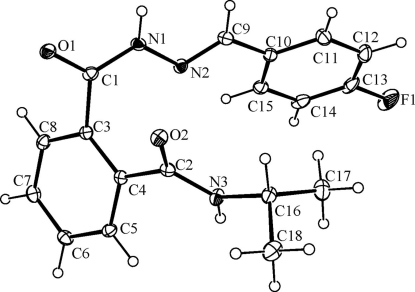
Molecular structure of (I); displacement ellipsoids are drawn at the 30% probability level. H atoms are shown as small circles of arbitrary radius.

**Fig. 2.**
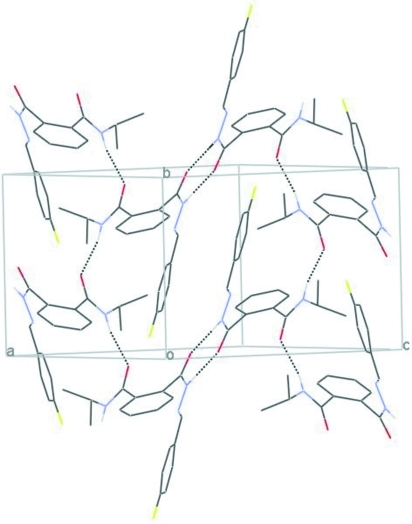
The crystal packing of (I) viewed along the [101] direction; hydrogen bonds are shown as dashed lines.

## supplementary crystallographic information

### Comment

Phthalic diamides possess insecticidal properties due to their ability to activate ryanodine receptor (Coronado *et al.*, 1994; Tohnishi *et al.*, 2000). The title compound (I), a new phthalic diamide derivative, was synthesized by the condensation of *N*-aminophthalimide with 4-fluorobenzaldehyde followed by a ring-opening reaction using isopropyl amine (Zaky, 2002; Shigeru *et al.*, 2003).

The molecular structure of the title compound is shown in Fig. 1. Molecule was proved to be a *trans *-isomer with respect to the C9=N2 double bond.

There are two independent N—H···O bonds (Table 1), which link molecules into the layers parallel to (101) plane (Fig. 2).

### Experimental

To a solution of *N*-aminophthalimide (1.62 g, 10 mmol) and 4-fluorobenzaldehyde (1.24 g, 10 mmol) in 1,4-dioxane (100 ml), 12 N HCl (0.1 ml) was added at room temperature. After stirring for 5–10 min, a solution of isopropyl amine (1.16 g, 20 mmol) in 1,4-dioxane (10 ml) was added; the reaction mixture was stirred overnight at room temperature. After the solvent was evaporated under reduced pressure, the resulting mixture was dissolved in ethyl acetate (80 ml), washed with H_2_O (3×30 ml) and dried with anhydrous sodium sulfate to give the title compound (2.01 g, 61.5%). Single crystals suitable for X-ray diffraction analysis were obtained by slow evaporation of ethanol solution at room temperature over one week.

### Refinement

The H atoms bound to N atoms were located in a difference Fourier map and refined isotropically [N—H 0.850 (15), 0.875 (15) Å]. The remaining H atoms were positioned geometrically and included in the refinement in riding model approximation with C—H 0.95 (aromatic), 0.98 (methyl), 1.00 (methyne), and *U*_iso_(H) = 1.2*U*_eq_(C)[1.5*U*_eq_(C) for methyl H atoms].

### Crystal data

**Table:**

C_18_H_18_FN_3_O_2_	*F*(000) = 688
*M**_r_* = 327.35	*D*_x_ = 1.303 Mg m^−^^3^
Monoclinic, *P*2_1_/*n*	Mo *K*α radiation, λ = 0.71073 Å
Hall symbol: -P 2yn	Cell parameters from 15335 reflections
*a* = 13.316 (3) Å	θ = 2.1–27.5°
*b* = 8.8904 (18) Å	µ = 0.09 mm^−^^1^
*c* = 14.102 (3) Å	*T* = 123 K
β = 91.10 (3)°	Block, colourless
*V* = 1669.2 (6) Å^3^	0.30 × 0.30 × 0.30 mm
*Z* = 4	

### Data collection

**Table:**

Rigaku R-AXIS RAPID IP diffractometer	3833 independent reflections
Radiation source: fine-focus sealed tube	2302 reflections with *I* > 2σ(*I*)
graphite	*R*_int_ = 0.046
Detector resolution: 10.00 pixels mm^-1^	θ_max_ = 27.5°, θ_min_ = 2.1°
Ω scans	*h* = −17→17
Absorption correction: multi-scan (*ABSCOR*; Higashi,1995)	*k* = −11→11
*T*_min_ = 0.944, *T*_max_ = 0.972	*l* = −18→18
15335 measured reflections	

### Refinement

**Table:**

Refinement on *F*^2^	Secondary atom site location: difference Fourier map
Least-squares matrix: full	Hydrogen site location: inferred from neighbouring sites
*R*[*F*^2^ > 2σ(*F*^2^)] = 0.039	H atoms treated by a mixture of independent and constrained refinement
*wR*(*F*^2^) = 0.069	*w* = 1/[σ^2^(*F*_o_^2^) + (0.015*P*)^2^] where *P* = (*F*_o_^2^ + 2*F*_c_^2^)/3
*S* = 1.02	(Δ/σ)_max_ < 0.001
3833 reflections	Δρ_max_ = 0.24 e Å^−^^3^
228 parameters	Δρ_min_ = −0.23 e Å^−^^3^
0 restraints	Extinction correction: *SHELXL97* (Sheldrick, 2008), Fc^*^=kFc[1+0.001xFc^2^λ^3^/sin(2θ)]^-1/4^
Primary atom site location: structure-invariant direct methods	Extinction coefficient: 0.0285 (8)

### Special details

**Table:**

Geometry. All e.s.d.'s (except the e.s.d. in the dihedral angle between two l.s. planes) are estimated using the full covariance matrix. The cell e.s.d.'s are taken into account individually in the estimation of e.s.d.'s in distances, angles and torsion angles; correlations between e.s.d.'s in cell parameters are only used when they are defined by crystal symmetry. An approximate (isotropic) treatment of cell e.s.d.'s is used for estimating e.s.d.'s involving l.s. planes.
Refinement. Refinement of *F*^2^ against ALL reflections. The weighted *R*-factor *wR* and goodness of fit *S* are based on *F*^2^, conventional *R*-factors *R* are based on *F*, with *F* set to zero for negative *F*^2^. The threshold expression of *F*^2^ > σ(*F*^2^) is used only for calculating *R*-factors(gt) *etc*. and is not relevant to the choice of reflections for refinement. *R*-factors based on *F*^2^ are statistically about twice as large as those based on *F*, and *R*- factors based on ALL data will be even larger.

### Fractional atomic coordinates and isotropic or equivalent isotropic displacement parameters (Å^2^)

**Table:**

	*x*	*y*	*z*	*U*_iso_*/*U*_eq_	
C1	0.62786 (10)	0.87434 (17)	0.48465 (10)	0.0197 (3)	
C2	0.70864 (10)	0.80962 (16)	0.29308 (10)	0.0179 (3)	
C3	0.72241 (10)	0.78555 (15)	0.47071 (10)	0.0168 (3)	
C4	0.76143 (10)	0.75512 (15)	0.38154 (10)	0.0162 (3)	
C5	0.85500 (10)	0.68578 (15)	0.37629 (10)	0.0197 (3)	
H5	0.8819	0.6634	0.3160	0.024*	
C6	0.90921 (11)	0.64909 (16)	0.45774 (11)	0.0228 (4)	
H6	0.9739	0.6050	0.4531	0.027*	
C7	0.86928 (10)	0.67651 (16)	0.54567 (11)	0.0236 (4)	
H7	0.9057	0.6494	0.6017	0.028*	
C8	0.77595 (10)	0.74366 (16)	0.55190 (10)	0.0216 (4)	
H8	0.7482	0.7613	0.6124	0.026*	
C9	0.44987 (11)	0.65483 (17)	0.36494 (10)	0.0222 (4)	
H9	0.3990	0.7297	0.3628	0.027*	
C10	0.43139 (10)	0.50842 (17)	0.32041 (10)	0.0215 (4)	
C11	0.35688 (11)	0.49509 (19)	0.24980 (11)	0.0299 (4)	
H11	0.3173	0.5803	0.2331	0.036*	
C12	0.33975 (12)	0.3594 (2)	0.20378 (12)	0.0384 (5)	
H12	0.2904	0.3510	0.1545	0.046*	
C13	0.39601 (13)	0.2385 (2)	0.23155 (12)	0.0366 (5)	
C14	0.46863 (12)	0.24391 (18)	0.30209 (11)	0.0307 (4)	
H14	0.5053	0.1565	0.3202	0.037*	
C15	0.48647 (11)	0.38099 (17)	0.34587 (11)	0.0237 (4)	
H15	0.5371	0.3882	0.3940	0.028*	
C16	0.66213 (10)	0.75633 (16)	0.12683 (10)	0.0221 (4)	
H16	0.6111	0.8369	0.1373	0.027*	
C17	0.60856 (12)	0.61917 (18)	0.08583 (11)	0.0326 (4)	
H17A	0.6574	0.5385	0.0758	0.049*	
H17B	0.5762	0.6458	0.0251	0.049*	
H17C	0.5576	0.5848	0.1301	0.049*	
C18	0.74105 (11)	0.81707 (18)	0.05991 (11)	0.0311 (4)	
H18A	0.7736	0.9053	0.0886	0.047*	
H18B	0.7087	0.8457	−0.0004	0.047*	
H18C	0.7915	0.7392	0.0486	0.047*	
F1	0.37958 (8)	0.10376 (12)	0.18747 (7)	0.0603 (4)	
N1	0.53997 (9)	0.82179 (14)	0.44990 (9)	0.0204 (3)	
N2	0.53387 (8)	0.68275 (13)	0.40673 (8)	0.0196 (3)	
N3	0.70850 (9)	0.71718 (14)	0.21866 (9)	0.0214 (3)	
O1	0.63114 (7)	0.99350 (11)	0.53105 (7)	0.0257 (3)	
O2	0.66866 (7)	0.93655 (11)	0.29200 (7)	0.0217 (3)	
H1	0.7381 (11)	0.6294 (17)	0.2210 (11)	0.038 (5)*	
H2	0.4879 (11)	0.8765 (18)	0.4542 (10)	0.039 (5)*	

### Atomic displacement parameters (Å^2^)

**Table:**

	*U*^11^	*U*^22^	*U*^33^	*U*^12^	*U*^13^	*U*^23^
C1	0.0210 (8)	0.0190 (8)	0.0193 (9)	−0.0014 (7)	0.0050 (6)	0.0005 (7)
C2	0.0172 (8)	0.0152 (8)	0.0215 (9)	−0.0017 (7)	0.0023 (6)	0.0018 (7)
C3	0.0184 (7)	0.0108 (7)	0.0214 (8)	−0.0035 (6)	0.0021 (6)	−0.0015 (6)
C4	0.0178 (8)	0.0110 (7)	0.0197 (8)	−0.0028 (6)	0.0009 (6)	0.0006 (7)
C5	0.0210 (8)	0.0172 (8)	0.0210 (9)	−0.0002 (7)	0.0043 (6)	−0.0024 (7)
C6	0.0191 (8)	0.0187 (8)	0.0305 (10)	0.0035 (7)	−0.0017 (7)	0.0004 (7)
C7	0.0258 (8)	0.0215 (8)	0.0233 (9)	−0.0006 (7)	−0.0052 (7)	0.0030 (7)
C8	0.0260 (8)	0.0222 (8)	0.0168 (8)	−0.0028 (7)	0.0026 (6)	−0.0006 (7)
C9	0.0184 (8)	0.0221 (9)	0.0262 (9)	0.0009 (7)	0.0017 (7)	0.0001 (7)
C10	0.0181 (8)	0.0267 (9)	0.0197 (9)	−0.0052 (7)	0.0038 (6)	−0.0024 (7)
C11	0.0228 (9)	0.0406 (10)	0.0264 (10)	−0.0045 (8)	−0.0005 (7)	−0.0008 (8)
C12	0.0292 (10)	0.0578 (13)	0.0284 (11)	−0.0181 (10)	0.0031 (8)	−0.0149 (10)
C13	0.0406 (11)	0.0358 (11)	0.0339 (11)	−0.0211 (9)	0.0180 (8)	−0.0213 (9)
C14	0.0332 (10)	0.0252 (9)	0.0343 (10)	−0.0042 (8)	0.0161 (8)	−0.0049 (8)
C15	0.0219 (8)	0.0259 (9)	0.0235 (9)	−0.0045 (7)	0.0056 (7)	−0.0009 (8)
C16	0.0262 (9)	0.0204 (8)	0.0197 (9)	0.0081 (7)	−0.0045 (7)	−0.0010 (7)
C17	0.0368 (10)	0.0280 (9)	0.0326 (10)	0.0038 (8)	−0.0088 (8)	−0.0045 (8)
C18	0.0387 (10)	0.0294 (9)	0.0253 (10)	0.0072 (8)	0.0010 (7)	0.0025 (8)
F1	0.0638 (7)	0.0548 (7)	0.0630 (8)	−0.0279 (6)	0.0211 (6)	−0.0401 (6)
N1	0.0160 (7)	0.0170 (7)	0.0283 (8)	0.0009 (6)	0.0017 (6)	−0.0049 (6)
N2	0.0213 (7)	0.0164 (6)	0.0211 (7)	−0.0029 (6)	0.0028 (5)	−0.0033 (6)
N3	0.0283 (8)	0.0166 (7)	0.0192 (7)	0.0067 (6)	−0.0036 (6)	−0.0018 (6)
O1	0.0229 (6)	0.0202 (6)	0.0340 (7)	−0.0007 (5)	0.0037 (5)	−0.0103 (5)
O2	0.0267 (6)	0.0135 (5)	0.0247 (6)	0.0028 (5)	0.0004 (5)	0.0015 (5)

### Geometric parameters (Å, °)

**Table:**

C1—O1	1.2455 (16)	C11—H11	0.9500
C1—N1	1.3438 (18)	C12—C13	1.363 (2)
C1—C3	1.5022 (19)	C12—H12	0.9500
C2—O2	1.2476 (16)	C13—F1	1.3656 (18)
C2—N3	1.3330 (18)	C13—C14	1.375 (2)
C2—C4	1.500 (2)	C14—C15	1.385 (2)
C3—C8	1.3879 (19)	C14—H14	0.9500
C3—C4	1.3964 (19)	C15—H15	0.9500
C4—C5	1.3934 (18)	C16—N3	1.4658 (18)
C5—C6	1.3836 (19)	C16—C17	1.521 (2)
C5—H5	0.9500	C16—C18	1.5247 (19)
C6—C7	1.380 (2)	C16—H16	1.0000
C6—H6	0.9500	C17—H17A	0.9800
C7—C8	1.3831 (18)	C17—H17B	0.9800
C7—H7	0.9500	C17—H17C	0.9800
C8—H8	0.9500	C18—H18A	0.9800
C9—N2	1.2786 (17)	C18—H18B	0.9800
C9—C10	1.464 (2)	C18—H18C	0.9800
C9—H9	0.9500	N1—N2	1.3796 (16)
C10—C15	1.393 (2)	N1—H2	0.850 (15)
C10—C11	1.3972 (19)	N3—H1	0.875 (15)
C11—C12	1.386 (2)		
			
O1—C1—N1	120.58 (13)	C11—C12—H12	121.1
O1—C1—C3	119.65 (13)	C12—C13—F1	118.60 (17)
N1—C1—C3	119.72 (13)	C12—C13—C14	123.75 (16)
O2—C2—N3	123.63 (14)	F1—C13—C14	117.65 (18)
O2—C2—C4	119.68 (13)	C13—C14—C15	117.74 (17)
N3—C2—C4	116.68 (13)	C13—C14—H14	121.1
C8—C3—C4	119.79 (13)	C15—C14—H14	121.1
C8—C3—C1	116.84 (13)	C14—C15—C10	121.07 (15)
C4—C3—C1	123.16 (13)	C14—C15—H15	119.5
C5—C4—C3	118.82 (13)	C10—C15—H15	119.5
C5—C4—C2	120.26 (13)	N3—C16—C17	109.39 (12)
C3—C4—C2	120.64 (12)	N3—C16—C18	110.32 (12)
C6—C5—C4	120.86 (14)	C17—C16—C18	111.86 (13)
C6—C5—H5	119.6	N3—C16—H16	108.4
C4—C5—H5	119.6	C17—C16—H16	108.4
C7—C6—C5	120.02 (14)	C18—C16—H16	108.4
C7—C6—H6	120.0	C16—C17—H17A	109.5
C5—C6—H6	120.0	C16—C17—H17B	109.5
C6—C7—C8	119.69 (14)	H17A—C17—H17B	109.5
C6—C7—H7	120.2	C16—C17—H17C	109.5
C8—C7—H7	120.2	H17A—C17—H17C	109.5
C7—C8—C3	120.76 (13)	H17B—C17—H17C	109.5
C7—C8—H8	119.6	C16—C18—H18A	109.5
C3—C8—H8	119.6	C16—C18—H18B	109.5
N2—C9—C10	120.58 (14)	H18A—C18—H18B	109.5
N2—C9—H9	119.7	C16—C18—H18C	109.5
C10—C9—H9	119.7	H18A—C18—H18C	109.5
C15—C10—C11	118.54 (15)	H18B—C18—H18C	109.5
C15—C10—C9	121.96 (14)	C1—N1—N2	121.06 (13)
C11—C10—C9	119.49 (14)	C1—N1—H2	118.7 (11)
C12—C11—C10	121.04 (16)	N2—N1—H2	120.2 (11)
C12—C11—H11	119.5	C9—N2—N1	114.89 (12)
C10—C11—H11	119.5	C2—N3—C16	122.92 (13)
C13—C12—C11	117.83 (16)	C2—N3—H1	121.8 (10)
C13—C12—H12	121.1	C16—N3—H1	115.3 (10)

### Hydrogen-bond geometry (Å, °)

**Table:**

*D*—H···*A*	*D*—H	H···*A*	*D*···*A*	*D*—H···*A*
N3—H1···O2^i^	0.875 (15)	2.127 (15)	2.9887 (16)	168.4 (14)
N1—H2···O1^ii^	0.850 (15)	1.976 (15)	2.8256 (16)	177.8 (15)

Symmetry codes: (i) −*x*+3/2, *y*−1/2, −*z*+1/2; (ii) −*x*+1, −*y*+2, −*z*+1.
